# Host metabolism dysregulation and cell tropism identification in human airway and alveolar organoids upon SARS-CoV-2 infection

**DOI:** 10.1007/s13238-020-00811-w

**Published:** 2020-12-12

**Authors:** Rongjuan Pei, Jianqi Feng, Yecheng Zhang, Hao Sun, Lian Li, Xuejie Yang, Jiangping He, Shuqi Xiao, Jin Xiong, Ying Lin, Kun Wen, Hongwei Zhou, Jiekai Chen, Zhili Rong, Xinwen Chen

**Affiliations:** Center for Biosafety Mega-Science, Wuhan Institute of Virology, Chinese Academy of Sciences, 430071, Wuhan, China; Cancer Research Institute, School of Basic Medical Sciences, Southern Medical University, 510515, Guangzhou, China; Center for Biosafety Mega-Science, Wuhan Institute of Virology, Chinese Academy of Sciences, 430071, Wuhan, China; Center for Biosafety Mega-Science, Wuhan Institute of Virology, Chinese Academy of Sciences, 430071, Wuhan, China; Cancer Research Institute, School of Basic Medical Sciences, Southern Medical University, 510515, Guangzhou, China; Guangzhou Institutes of Biomedicine and Health, Chinese Academy of Sciences, 510530, Guangzhou, China; Joint School of Life Sciences, Guangzhou Medical University and Guangzhou Institutes of Biomedicine and Health, Chinese Academy of Sciences, 511436, Guangzhou, China; Guangzhou Institutes of Biomedicine and Health, Chinese Academy of Sciences, 510530, Guangzhou, China; The Centre of Cell Lineage and Atlas (CCLA), Bioland Laboratory (Guangzhou Regenerative Medicine and Health-Guangdong Laboratory), 510530, Guangzhou, China; Center for Biosafety Mega-Science, Wuhan Institute of Virology, Chinese Academy of Sciences, 430071, Wuhan, China; Center for Biosafety Mega-Science, Wuhan Institute of Virology, Chinese Academy of Sciences, 430071, Wuhan, China; Cancer Research Institute, School of Basic Medical Sciences, Southern Medical University, 510515, Guangzhou, China; Microbiome Medicine Center, Division of Laboratory Medicine, Zhujiang Hospital, Southern Medical University, Guangzhou, China; Microbiome Medicine Center, Division of Laboratory Medicine, Zhujiang Hospital, Southern Medical University, Guangzhou, China; Guangzhou Institutes of Biomedicine and Health, Chinese Academy of Sciences, 510530, Guangzhou, China; The Centre of Cell Lineage and Atlas (CCLA), Bioland Laboratory (Guangzhou Regenerative Medicine and Health-Guangdong Laboratory), 510530, Guangzhou, China; Joint School of Life Sciences, Guangzhou Medical University and Guangzhou Institutes of Biomedicine and Health, Chinese Academy of Sciences, 511436, Guangzhou, China; Cancer Research Institute, School of Basic Medical Sciences, Southern Medical University, 510515, Guangzhou, China; Bioland Laboratory (Guangzhou Regenerative Medicine and Health Guangdong Laboratory), 510005, Guangzhou, China; Dermatology Hospital, Southern Medical University, 510091, Guangzhou, China; Center for Biosafety Mega-Science, Wuhan Institute of Virology, Chinese Academy of Sciences, 430071, Wuhan, China; Guangzhou Institutes of Biomedicine and Health, Chinese Academy of Sciences, 510530, Guangzhou, China

**Keywords:** COVID-19, SARS-CoV-2, lung organoids, cell tropism, cellular metabolism, drug discovery

## Abstract

The coronavirus disease 2019 (COVID-19) pandemic is caused by infection with the severe acute respiratory syndrome coronavirus 2 (SARS-CoV-2), which is spread primary via respiratory droplets and infects the lungs. Currently widely used cell lines and animals are unable to accurately mimic human physiological conditions because of the abnormal status of cell lines (transformed or cancer cells) and species differences between animals and humans. Organoids are stem cell-derived self-organized three-dimensional culture *in vitro* and model the physiological conditions of natural organs. Here we showed that SARS-CoV-2 infected and extensively replicated in human embryonic stem cells (hESCs)-derived lung organoids, including airway and alveolar organoids which covered the complete infection and spread route for SARS-CoV-2 within lungs. The infected cells were ciliated, club, and alveolar type 2 (AT2) cells, which were sequentially located from the proximal to the distal airway and terminal alveoli, respectively. Additionally, RNA-seq revealed early cell response to virus infection including an unexpected downregulation of the metabolic processes, especially lipid metabolism, in addition to the well-known upregulation of immune response. Further, Remdesivir and a human neutralizing antibody potently inhibited SARS-CoV-2 replication in lung organoids. Therefore, human lung organoids can serve as a pathophysiological model to investigate the underlying mechanism of SARS-CoV-2 infection and to discover and test therapeutic drugs for COVID-19.

## INTRODUCTION

The current fast-evolving coronavirus disease 2019 (COVID-19) pandemic is caused by the severe acute respiratory syndrome coronavirus 2 (SARS-CoV-2), which infects lungs and can lead to severe lung injury, multiorgan failure, and death (Li et al., 2020; Wiersinga et al., 2020; Zhu et al., 2020). To prevent and effectively manage COVID-19, public health, clinical interventions, and basic and clinical research are all emergently required. For basic research, it is essential to establish models that can faithfully reproduce the viral life cycle and mimic the pathology of COVID-19.

**Figure 1:**
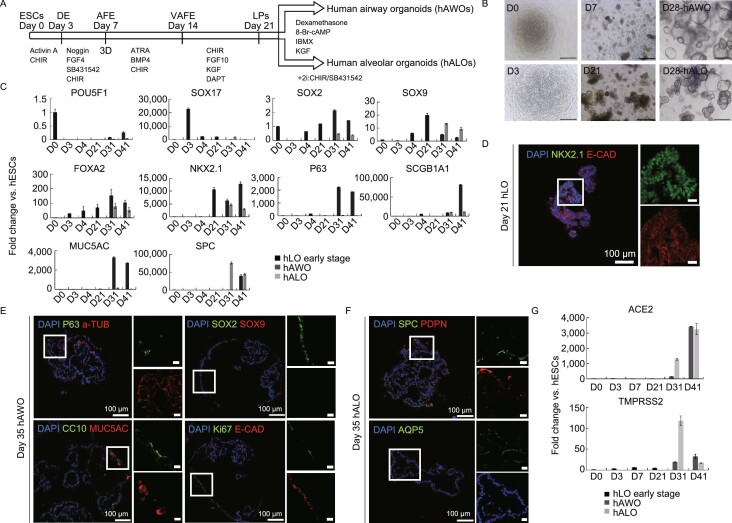
**Generation of human airway and alveolar organoids from hESCs**. (A) Schematic of differentiation protocol and stages from hESCs to human airway organoids (hAWOs) and human alveolar organoids (hALOs). (B) Representative images at the indicated differentiation stages. Scale bar, 500 μm. (C) Fold change of lineage marker genes from day 0 (D0) to D41 over undifferentiated hESCs by quantitative RT-PCR (2^−ΔΔCt^). D0–D21, hLOs early stage. D21–D41, organoids split into two groups with different differentiated medium (hAWOs and hALOs). *POU5F1*, embryonic stem cell marker, *SOX17*, definitive endoderm marker, *SOX2*, embryonic stem cell and proximal airway cell marker, *SOX9*, distal alveolar progenitor cell marker, *FOXA2* and *NKX2.1*, lung progenitor lineage marker, *P63*, basal cell marker, *SCGB1A1* (*CC10*), club cell marker, *MUC5AC*, goblet cell marker, *SPC*, AT2 cell marker. Normalized to *GAPDH*. Bars represent mean ± SD, *n* = 3. (D–F) Cell lineage marker expression in human lung progenitor organoids (hLOs), human airway organoids (hAWOs), and human alveolar organoids (hALOs). Immunofluorescence images of NKX2.1 and E-Cadherin (epithelial cells) expression in D21 hLOs (D), of P63, SOX2, CC10, Ki67 (proliferation cells) and acetylated tubulin (ciliated cells), SOX9, MUC5AC, E-Cadherin protein expression in D35 hAWOs (E), and of SPC, AQP5 (AT1) and PDPN (AT1) expression in D35 hALOs (F). Nuclei were counterstained with DAPI. Scale bar, 100 μm (left panel); 20 μm (right panel). Boxes represent zoom views. (G) Fold change of *ACE2* and *TMPRSS2* gene expression from D0 to D41 over undifferentiated hESCs by quantitative RT-PCR (2^−ΔΔCt^). Normalized to *GAPDH*. Bars represent mean ± SD, *n* = 3

**Figure 2:**
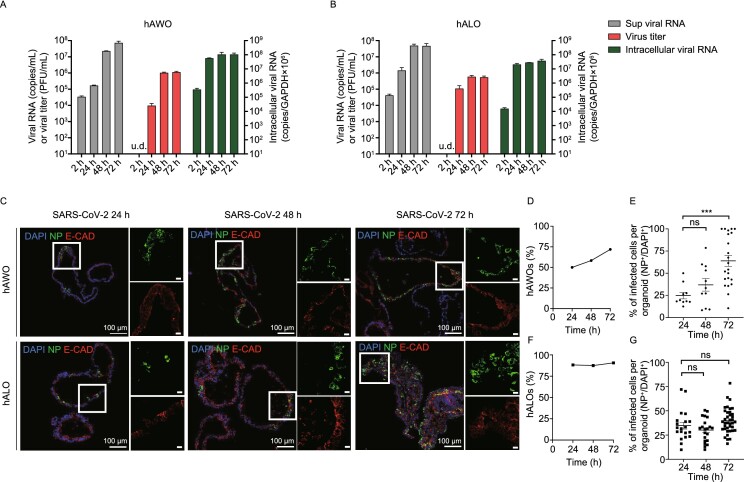
**SARS-CoV-2 replicates in human airway and alveolar organoids**. (A and B) The viral RNA and virus titer in the culture supernatant and relative intracellular viral RNA in cell lysates in hAWOs (A) and hALOs (B) were detected at indicated time points post infection. (C) Immunofluorescence images of viral nucleoprotein (green) and epithelial marker E-cadherin (red) expression with DNA stain (DAPI, blue) in SARS-CoV-2 infected hAWOs and hALOs. Scale bar, 100 μm (left panel); 20 μm (right panel). Boxes represent zoom views. (D and F) Percentage of hAWOs (D) and hALOs (F) harboring SARS-CoV2 infected cells at different time points. At least 30 different organoids were counted per condition. (E and G) Percentage of infected cells per infected hAWOs (E) and hALOs (G). At least 10 organoids were counted in (E) and at least 20 organoids in (G). ***P < 0.001, by one-way ANOVA analysis

**Figure 3:**
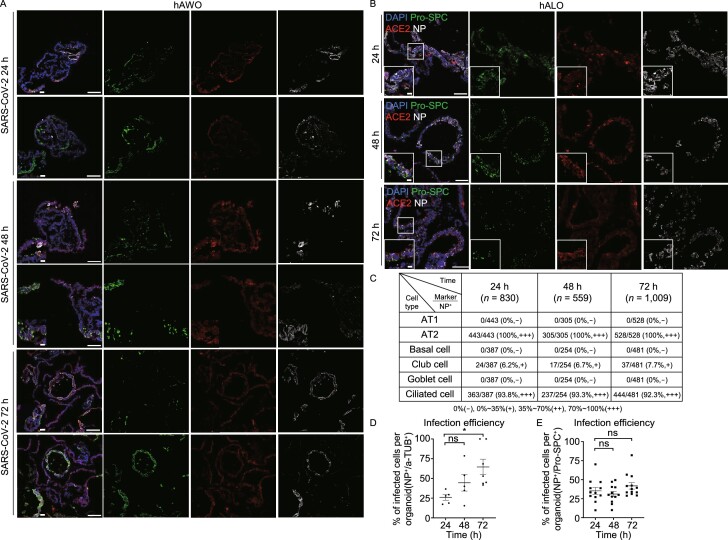
**SARS-CoV-2 Infects ciliated, club, and alveolar type 2 cells**. (A and B) Representative immunofluorescence images of nucleoprotein, ACE2 and indicated cell linage marker expression with DNA stain (DAPI). Club cells (CC10^+^) and ciliated cells (acetylated Tubulin^+^) were stained in human airway organoids at indicated time points (A). Arrowheads indicate infected club cells. Alveolar type 2 cells (pro-SPC^+^) were stained in human alveolar organoids (B). Scale bars, 100 µm; bottom left corner, 20 µm. Boxes represent zoom views. (C) Summary of the percentage of different cell types to SARS-CoV-2 infected cells in human airway and alveolar organoids. 830, 559, and 1,009 nucleoprotein positive cells were counted at 24, 48, and 72 hpi, respectively. − negative for the nucleoprotein staining; +, 0%–35% positive for the nucleoprotein; ++, 35%–70% positive for the nucleoprotein; +++, 70%–100% positive for the nucleoprotein. (D and E) Percentage of infected ciliated cells (acetylated Tubulin^+^) per infected airway organoid (D) and infected alveolar type 2 cells (pro-SPC^+^) per alveolar organoid (E). At least 5 organoids were counted in D and at least 13 organoids in E. * *P* < 0.05, by one-way ANOVA analysis

**Figure 4:**
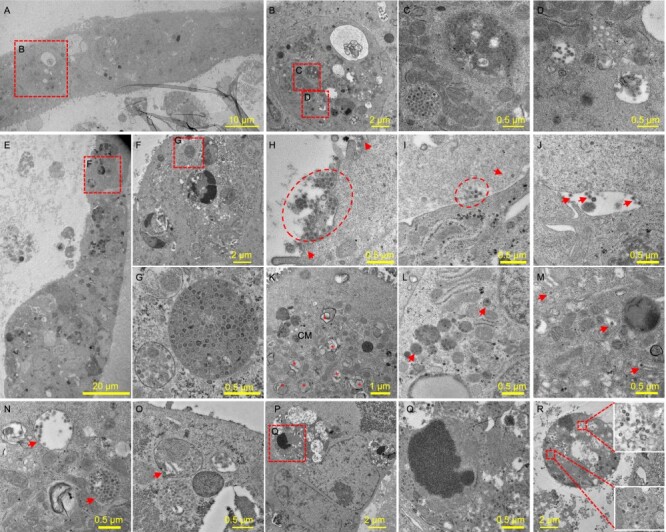
**Transmission electron microscopy analysis of SARS-CoV-2 infected human airway and alveolar organoids**. (A–D) Infected hAWOs were fixed and observed under TEM at 96 h post infection. A part of the organoids in one mesh was overviewed (A) and the virus particles in an infected cell were shown (B–D). (E–G) Infected hALOs were fixed at 72 h post infection. A part of the organoids in one mesh (E) and the virus particles in an infected cell (F and G) were shown. (H–R) Representative virus particles and typical structures induced by virus infection in hAWOs (H–N) and hALOs (O–R). Virus particles outside cells at the apical (H), basolateral (I) and lateral side (J). Typical coronavirus replication organelle including double membrane vesicles (DMVs, indicated by asterisks) and convoluted membranes (CMs) with spherules (K). Membrane-bound vesicles with one or groups of virus particles (L). Enveloped virus particles in Golgi apparatus (M). Enveloped virus particles in secretory vesicles (N). Virus particles in a lamella body (O). Virus particles in a late endosome with engulfed cell debris (P and Q). Virus particles in disintegrated dead cells (R)

**Figure 5:**
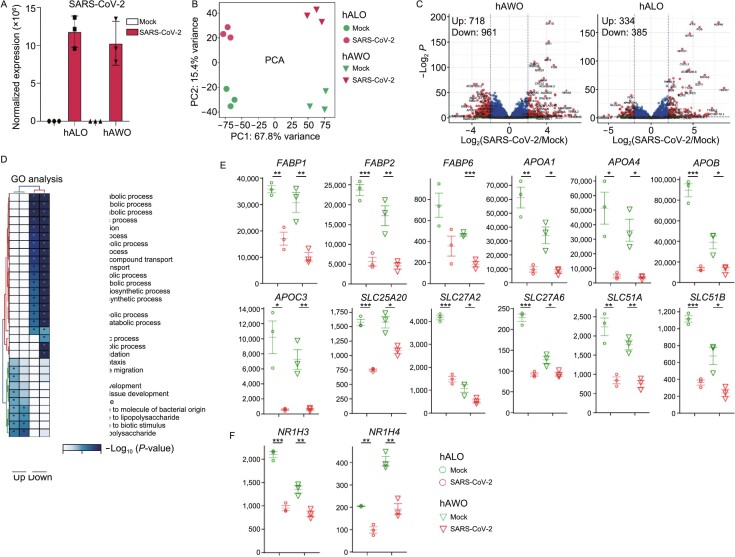
**SARS-CoV-2 infection downregulates metabolic processes in human lung organoids**. (A) SARS-CoV-2 viral RNA detected by RNA-seq in mock and infected organoids harvested at 48 hpi. Data are expressed as normalized read counts. (B) PCA plot for the Mock and SARS-CoV-2 infected organoids. (C) Volcano plot showing differentially expressed genes in the SARS-CoV-2 infected organoids compared with mock control. (D) Gene ontology (GO) analysis showing the differentially expressed genes from panel (C). (E) Expression level of lipid metabolism related genes, the grey lines are the means of the three biological replicates, and the error bars are the standard error of the mean. Data are expressed as normalized read counts. *P*-values are from a one-tailed Student's *t* test. *P < 0.05, **P < 0.01, ***P < 0.001, ****P < 0.0001. (F) Expression level of LXRα (also known as NR1H3) and FXR (also known as NR1H4), two critical nuclear receptors regulating sterol, fatty acid and glucose metabolism

**Figure 6:**
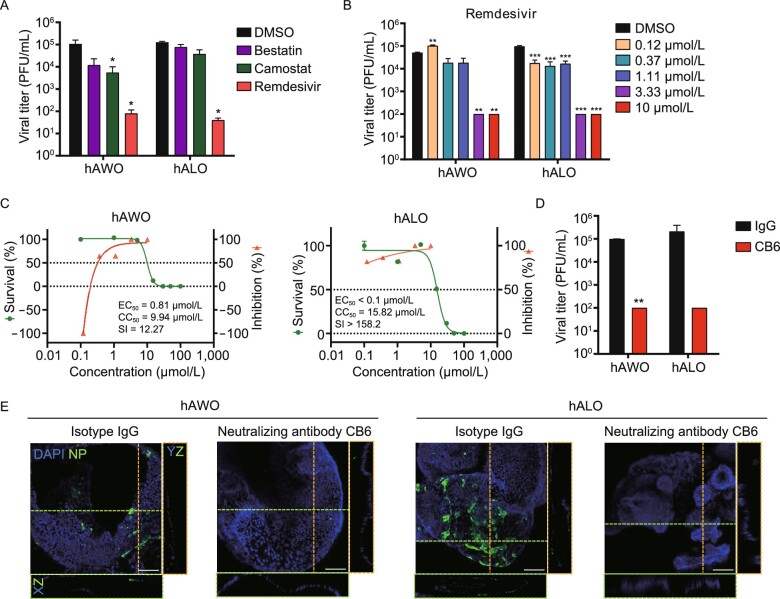
**Remdesivir and a human neutralizing antibody inhibit SARS-CoV-2 replication in lung organoids**. (A) hAWOs and hALOs were infected with SARS-CoV-2, the indicated compounds were added into the culture media 2 h after infection. 48 h later, the virus titers were determined by plaque assay with Vero E6 cells. *P < 0.05, by one-way ANOVA analysis. (B) Virus infected hAWOs and hALOs were treated with remdesivir at indicated concentrations for 48 h. The virus titers were determined by plaque assay. **P < 0.01, ***P < 0.001, by one-way ANOVA analysis. (C) Inhibition and toxicity curves of remdesivir in hAWOs and hALOs. Inhibition and cytotoxicity of remdesivir was quantified by viral titers and viable cell counting, respectively. The left and right *Y-axis* of these graphs represent mean survival of the cells and inhibition of virus titers, respectively. Bars represent mean ± SD, *n* = 3. (D and E) hAWOs and hALOs were infected with SARS-CoV-2 at the present of a human neutralizing antibody CB6 or isotype IgG, and virus titers were detected at 48 hpi. **P < 0.01, by unpaired, two-tailed Student's *t* test (D). Whole-mount staining of hAWOs and hALOs. Nucleoprotein (NP) was stained to visualize infected cells. The XZ and YZ planes of the horizontal and vertical cut view of Z-stack images are shown at the bottom and right, respectively (E). Scale bars: 100 µm

Cell lines and animals are two major models for coronavirus infection *in vitro* and *in vivo*, respectively (Kaye, 2006; Song et al., 2019; Hoffmann et al., 2020; Takayama, 2020). Cell lines can be used to amplify and isolate viruses (like Vero and Vero E6 cells) (Harcourt et al., 2020; Zhou et al., 2020b), to investigate the viral infection (like primary human airway epithelial cells, Caco-2 and Calu-3 cells) (Hoffmann et al., 2020; Kim et al., 2020; Ou et al., 2020; Zhu et al., 2020), and to evaluate therapeutic molecules (like Huh7 and Vero E6 cells) (Wang et al., 2020a). Animal models can be used to mimic tissue-specific and systemic virus-host interaction and reveal the complex pathophysiology of coronaviruses-induced diseases (Song et al., 2019). Mice, hamster, ferrets, cats, and non-human primates have been reported to model COVID-19 (Bao et al., 2020; Chandrashekar et al., 2020; Jiang et al., 2020; Rockx et al., 2020; Shi et al., 2020a; Sia et al., 2020; van Doremalen et al., 2020; Williamson et al., 2020; Yu et al., 2020). These cell and animal models have greatly enriched our understanding of coronaviruses and assisted in the development of a variety of potential therapeutic drugs (Song et al., 2019). However, these models yet have obvious limitations. Species differences make animal model results unable to be effectively translated into clinical applications (Martic-Kehl et al., 2012; Warren et al., 2015). Species differences (cells from species other than humans, like Vero cells) and abnormal status (transformed or cancer cells) make cell models unable to faithfully reproduce the viral infection cycle and host response (Sun et al., 2002; Pan et al., 2009; Cairns et al., 2011).

Organoids are a three-dimensional structure formed by self-assembly of stem cells *in vitro* (Clevers, 2016; Rossi et al., 2018). As the cell composition, tissue organization, physiological characteristics, and even functions are similar to natural organs in the body, organoids have been used for human virus studies (Dutta and Clevers, 2017; Ramani et al., 2018). For SARS-CoV-2 study, kidney, liver, intestine, and blood vessel organoids have been documented (Lamers et al., 2020; Monteil et al., 2020; Yang et al., 2020; Zhao et al., 2020; Zhou et al., 2020a). Here using human embryonic stem cells (hESCs)-derived lung airway and alveolar organoids, we demonstrated that SARS-CoV-2 infects ciliated, club, and alveolar type 2 (AT2) cells, and that downregulation of metabolic processes, particularly lipid metabolism, was another featured cell response to virus infection in addition to the well-known immune response. Further, we also proved that Remdesivir and a human neutralizing antibody potently inhibited SARS-CoV-2 replication in lung organoids.

## RESULTS

### Generation of human lung airway and alveolar organoids from hESCs

Based on our previous protocol (Chen et al., 2018), as well as other reported protocols (McCauley et al., 2017; Yamamoto et al., 2017), we developed an optimized method to differentiate human airway organoids (hAWOs) and alveolar organoids (hALOs) from hESCs, which contained six stages, embryonic stem cells (ESCs), definitive endoderm (DE), anterior foregut endoderm (AFE), ventralized anterior foregut endoderm (VAFE), lung progenitors (LPs), and hAWOs and hALOs (Fig. 1A and 1B). Quantitative RT-PCR revealed the expression dynamics of marker genes along differentiation (Fig. 1C). *POU5F1* (ESCs), *SOX17* (DE), *SOX2* (ESCs and lung proximal progenitors), *SOX9* (lung distal progenitors), *FOXA2* (lung epithelial cells), *NKX2.1* (lung epithelial cells), *P63* (basal cells), *SCGB1A1* (club cells), *MUC5AC* (goblet cells) and *SPC* (AT2 cells) showed expected expression patterns (Fig. 1C). Human lung organoids (hLOs) at day 21 (D21) expressed lung and pan epithelial markers NKX2.1 and E-CAD, respectively (Fig. 1D). Immunofluorescent staining revealed that hAWOs contained basal cells (P63^+^), ciliated cells (acetylated TUBULIN, a-TUB^+^), club cells (CC10^+^), and goblet cells (MUC5AC^+^), as well as lung proximal progenitors (SOX2^+^) and proliferating cells (Ki67^+^) (Fig. 1E). And hALOs contained AT2 cells (SPC^+^) and AT1 cells (PDPN^+^ or AQP5^+^) (Fig. 1F). Since ACE2 is the receptor for SARS-CoV-2 for host cell entry and TMPRSS2 is the serine protease for spike (S) protein priming (Hoffmann et al., 2020; Zhou et al., 2020b), we checked their expression along the differentiation and found they were highly expressed in hAWOs and hALOs (Fig. 1G).

### SARS-CoV-2 infects human airway and alveolar organoids

To test whether SARS-CoV-2 infects human lung organoids, hAWOs and hALOs (ranging from D31 to D41) were exposed to SARS-CoV-2 at a multiplicity of infection (MOI) of 1. Samples were harvested at indicated time points after infection and processed for the various analyses shown in Figures 2–5. Live virus titration on Vero E6 cells and quantitative RT-PCR of viral RNA in the culture supernatant and cell lysates showed that hAWOs and hALOs were productively infected by SARS-CoV-2 (Fig. 2A and 2B). Viral RNA and infectious virus particles could be detected as early as 24 h post infection (hpi), increased at 48 hpi, and remained stable at 72 hpi. Compared to hALOs, hAWOs produced less virus at 24 hpi and similar amount of virus at 48 hpi and 72 hpi (Fig. 2A and 2B). Co-immunostaining of viral nucleocapsid protein (NP) and pan epithelial marker E-CAD showed that SARS-CoV-2 infected epithelial cells in human lung organoids (Fig. 2C). Quantification analysis showed that the percentages of infected hAWOs increased from about 50% at 24 hpi to about 75% at 72 hpi (Fig. 2D). And the percentages of infected cells within a single hAWO increased from about 24.9% ± 3.7% at 24 hpi to 63.9% ± 6.1% at 72 hpi (Fig. 2E). For hALOs, the percentages of infected organoids remained stable at about 85% and the percentages of infected cells per organoid remained about 30%–40% from 24 hpi to 72 hpi. These cellular infection results were consistent with viral RNA detection and infectious viral particle titration results.

### SARS-CoV-2 infects ciliated, club, and alveolar type 2 cells

To determine the cell tropism of SARS-CoV-2, we co-stained each cell lineage marker with viral N protein (NP) and the virus receptor ACE2. Microscopy analyses revealed that ciliated cells (a-TUB^+^) and alveolar type 2 cells (Pro-SPC^+^) were the major target cells (Figs. 3A, 3B, and S1), which was consistent with the previous report (Hou et al., 2020). In addition, a subpopulation of club cells (CC10^+^) could be infected (Fig. 3A). In hAWOs, about 90%–95% infected cells were ciliated cells and about 5%–10% were club cells, and no basal (P63^+^) or goblet cells (MUC5AC^+^) were found infected (Fig. 3C). In hALOs, 100% infected cells were AT2 cells and no AT1 cells (PDPN^+^) were found infected (Fig. 3C). We also measured the percentages of infected cells within ciliated cells and AT2 cells. About 26% ± 3.6% at 24 hpi and 64.5% ± 9.8% at 72 hpi of ciliated cells were infected, and the percentages of infected AT2 cells remained stable at about 30%–40% from 24 hpi to 72 hpi (Fig. 3D and 3E). The distinct infection dynamics of ciliated cells and AT2 cells indicated that more and more ciliated cells could be infected by SARS-CoV-2 during a prolonged infection period and even all the ciliated cells could be finally infected when given long enough infection time. On the contrary, only a subpopulation of AT2 cells (about 30%–40%) was sensitive for viral infection although they could be quickly infected (within 24 hpi). The identity of the SARS-CoV-2 sensitive AT2 cell subpopulation and why other AT2 cells could not be infected need further investigation.

We noted that the infected cells expressed ACE2 but not all ACE2 expressing cells were infected. TMPRSS2 is another known factor that determines SARS-CoV-2 cell entry (Hoffmann et al., 2020), and therefore we checked the expression pattern of TMPRSS2 in human lung organoids. Immunostaining analyses showed that TMPRSS2 was ubiquitously expressed in both hAWOs and hALOs, which was contrary to the restricted expression pattern of ACE2 (Fig. S2). Therefore, these data imply that ACE2 is required for SARS-CoV-2 infection in human lung organoids as well as that host factors other than TMPRSS2 might facilitate SARS-CoV-2 cell entry and infectivity, like Neuropilin-1 and other unidentified factors (Cantuti-Castelvetri et al., 2020; Daly et al., 2020).

Next, we checked whether SARS-CoV-2 infection was associated with proliferation status by co-immunostaining with viral N protein and Ki67 (cycling marker). We found that infected cells (NP^+^) contained both cycling (Ki67^+^) and noncycling (Ki67^−^) cells in hAWOs and most infected cells were cycling cells in hALOs (Fig. S3A). We then checked whether SARS-CoV-2 infection induced apoptosis by co-immunostaining with NP and cleaved Caspase3 (C-Caspas3, apoptotic cell marker). No obvious cell death was observed at 24 hpi or 48 hpi, but at 72 hpi, apoptosis became prominent in both organoids, particularly more in hALOs (Fig. S3B–D).

### Characteristics of SARS-CoV-2 replication in human lung organoids

To confirm the viral replication, the ultrastructures of infected hAWOs and hALOs were analyzed by transmission electron microscopy (TEM) at 72 hpi or 96 hpi. Part of hAWOs and hALOs in one mesh of the grids were shown in Fig. 4A and 4E, and viral particles were found in cells of both organoids (Fig. 4B–D, 4F and 4G). In both organoids, viral particles were observed in the apical, lateral and basolateral side of the cells (Fig. 4H–J), indicating potential dissemination route how SARS-CoV-2 passes across the lung epithelial barrier. Double membrane vesicles (DMVs) and convoluted membranes (CMs) with spherules are typical coronavirus replication organelles (van Hemert et al., 2008; Hilgenfeld and Peiris, 2013), which were observed in the lung organoids (Fig. 4K). Virus particles in cells were seen in membrane bound vesicles, either as single particles or as groups in enlarged vesicles (Fig. 4L). Enveloped viruses were observed in the lumen of Golgi apparatus and secretory vesicles (Fig. 4M and 4N), which was consistent with previous report that coronaviruses assembled and matured at the endoplasmic reticulum-Golgi intermediate compartment (ERGIC) and the mature virions were transported to the cell surface and released from the host cells via exocytosis (Hilgenfeld and Peiris, 2013; Fehr and Perlman, 2015). Therefore, TEM analyses captured three critical phases of SARS-CoV-2 life cycle: replication, assembly and release.

Interestingly, we found virus particles within lamellar bodies (Fig. 4O), the typical organelles in AT2 cells, which are essential for pulmonary surfactant synthesis and secretion (Schmitz and Muller, 1991). Does SARS-CoV-2 hijack lamellar bodies for virus release? Or does SARS-CoV-2 impair the function of lamellar bodies and then the homeostasis of pulmonary surfactant in the alveoli? These questions remain open for further investigation. Additionally, vesicles full of dense virus particles were routinely observed (Fig. 4B, 4G and 4N). Besides, virus particles were found in late endosomes with engulfed cell debris (Fig. 4P and 4Q). And more dying cells and engulfed cell debris were observed in hALOs than in hAWOs (Fig. 4R). The TEM data (Fig. 4P–R), as well as the C-Caspase3 immunostaining data (Fig. S3B–D), indicated that the pathological changes of alveoli and bronchioles after SARS-CoV-2 infection were different.

### Early cell response to SARS-CoV-2 infection

To determine the early cell response to SARS-CoV-2 infection, we performed RNA-sequencing analysis using hAWOs and hALOs at 48 hpi. Abundant SARS-CoV-2 viral RNA was detected solely in the infected organoids (Fig. 5A). Principle component analysis (PCA) showed that the samples formed four separate clusters according to organoid type and virus infection (Fig. 5B). In total, 1,679 differential expressed genes were identified with 718 genes upregulated and 961 genes downregulated in hAWOs, and 719 genes differential expressed in hALOs with 334 upregulated and 385 downregulated (Fig. 5C). Gene ontology (GO) analysis revealed that most downregulated genes were associated with cell metabolism, especially lipid metabolism, while upregulated genes were associated with immune response (Fig. 5D). Several cytokines and chemokines, including interleukin (IL)-6, tumor necrosis factor (TNF), *CXCL8, CXCL2, CXCL3, CXCL10, CXCL11*, as well as NF-kB related mRNA *NFKB1, NFKB2* and *RELB,* interferon-stimulated genes *ATF3, GEM, IFITM3* and *MX1* were upregulated, consistent with previous observation in COVID-19 patients (Huang et al., 2020; Suzuki et al., 2020; Wilk et al., 2020) (Fig. S4A). Of note, TNF is a well-known inducer for virus-induced cell death (Zhou et al., 2017) and the increased mRNA expression level of TNF at 48 hpi might contribute to cell death at 72 hpi (Fig. S3B–D).

Fatty acid-binding proteins (FABPs) bind and transfer lipid between intracellular and extracellular membranes, and thus regulate fatty acid import, storage and export as well as phospholipid and cholesterol metabolism (Furuhashi and Hotamisligil, 2008; Hotamisligil and Bernlohr, 2015). Apolipoproteins (Apo) are structural components of lipoprotein particles and guide lipoprotein formation, function as ligands for lipoprotein receptors, and act as cofactors for enzymes involved in lipoprotein metabolism (Feingold and Grunfeld, 2000). The solute carrier (SLC) group of membrane transport proteins form a huge family and different subfamilies serve unique functions. For example, SLC25A20 transports the fatty acids carnitine and acylcarnitine across the mitochondrial inner membrane (Ruprecht and Kunji, 2020), SLC27 subfamily members mediate the uptake and activation of long chain fatty acids (LCFA) (Anderson and Stahl, 2013), and SLC51A and SLC51B form a heterodimer to export or uptake bile acids and steroids (Dawson et al., 2010). Members of all the three families play essential roles in lipid metabolism and were found downregulated in SARS-CoV-2 infected human airway and alveolar organoids, including FABP1/2/6, APOA1/4, APOB, APOC4, SLC25A20, SLC27A2/6, and SLC51A/B (Fig. 5E). Triacylglycerols (TAG) represent the predominant form of storage and transport of fatty acids within cells and in the plasma. Monoacylglycerol acyltransferases (MOGATs), lipin phosphatidic acid phosphatases (LIPINs), and diacylglycerol acyltransferases (DGATs) are key enzymes in TAG biosynthesis (Wang et al., 2017), and all the three types of enzymes were downregulated upon infection, including MOGAT1/2/3, LPIN3, and DGAT1/2 (Fig. S4B). Acting as transcription factors, nuclear receptors integrate hormonal and nutritional signals and orchestrate cellular metabolism. The liver X receptor α (LXRα, also known as NR1H3) and the farnesoid X receptor (FXR, also known as NR1H4) interact with the retinoid X receptor (RXR) and play essential roles in fatty acid, cholesterol, sterol, bile acid and glucose metabolism (Calkin and Tontonoz, 2012), and both were observed downregulated upon infection (Fig. 5F). In addition, metabolic reaction enrichment analysis (MaREA) using the MaREA4Galaxy tool can generate a metabolic atlas (Damiani et al., 2020). SARS-CoV-2 infection downregulated urea cycle in airway organoids and downregulated folate metabolism, glutamine metabolism and urea cycle in alveolar organoids (Fig. S4D and S4E).

ACE2 is the receptor for SARS-CoV-2 cell entry. We found that the mRNA expression level of *ACE2* was downregulated at 48 h after SARS-CoV-2 infection (Fig. S4C). Since most infected cells were viable at 48 hpi (Fig. S3B–D), the downregulation of *ACE2* mRNA was not a secondary effect of cell death but a direct effect of virus infection. Spike (S) proteins of SARS-CoV and SARS-CoV-2 has been reported to induce shedding of ACE2 by TMPRSS2 or ADAM17, which is believed to be a crucial mechanism for virus-induced pathogenesis (Kuba et al., 2005; Glowacka et al., 2010; Heurich et al., 2014; Banu et al., 2020; Vaduganathan et al., 2020; Verdecchia et al., 2020; Xiao et al., 2020). Therefore, we believe that SARS-CoV-2 infection might decrease the expression of *ACE2* at both protein and mRNA levels. However, the mechanisms of mRNA downregulation remain open for further investigation. In addition, we found that the expression of *TMPRSS2* was also slightly downregulated after SARS-CoV-2 infection at a much less extent than *ACE2* (Fig. S4C).

### Drug discovery using human lung organoids

Finally, we tested the inhibitory effect of small molecules and neutralizing antibodies on the infection of human lung organoids by SARS-CoV-2. Remdesivir is a nucleotide analogue prodrug to inhibit viral replication (Eastman et al., 2020), which has been reported to repress SARS-CoV-2 infection in basic research and clinic trials (Beigel et al., 2020; Wang et al., 2020a; Wang et al., 2020b). Camostat is an inhibitor of the serine protease TMPRSS2 that cleaves SARS-CoV-2 S protein and facilitates viral entry (Hoffmann et al., 2020). Bestatin is an inhibitor of CD13 (Aminopeptidase N/APN) (Jia et al., 2010), a receptor utilized by many α-coronaviruses (SARS-CoV-2 belongs to β-coronaviruses) (Fehr and Perlman, 2015). As shown in Fig. 6A, remdesivir reduced the production of infectious viral particles in hAWOs and hALOs, and camostat showed a slightly inhibitory effect in hAWOs not in hALOs, while bestatin had no effects in either hAWOs or hALOs. Quantitative RT-PCR analyses of supernatant viral RNA also demonstrated that remdesivir inhibited viral load (Fig. S5A). Whole-mount immunostaining assay also revealed the inhibitory effect of remdesivir (Fig. S5B). Dose-dependent assay further confirmed the repressive effect of remdesivir on virus titer (Fig. 6B). To quantify the efficacy of remdesivir, the inhibitory effect and the cytotoxicity was determined by virus titer assay and viable cell counting assay, respectively. The results showed that the half-maximal effective concentration (EC_50_) was 0.81 μmol/L, halfcytotoxic concentration (CC_50_) was 9.94 μmol/L, and selectivity index (SI) was 12.27 in airway organoids, and EC_50_ was less than 0.1 μmol/L, CC_50_ was 15.82 μmol/L, and SI was more than 158.2 in alveolar organoids (Fig. 6C).

Neutralizing antibodies are promising molecules to protect against SARS-CoV-2 and treat COVID-19. Therefore, we tested the inhibitory effect of a neutralizing antibody CB6 on virus infection in our human lung organoids. CB6 has been reported to inhibit SARS-CoV-2 infection in rhesus monkeys (Shi et al., 2020b). Similarly, CB6 significantly repressed the production of infectious viral particles in human lung organoids (Fig. 6D), and whole-mount immunostaining confirmed the protection effect (Fig. 6E). In summary, all the above results demonstrated that human lung organoids could serve as a platform to discover and test therapeutic drugs for COVID-19.

## DISCUSSION

In our study, both airway and alveolar organoids were developed and investigated, likely covering the complete infection and spread route for SARS-CoV-2 within lungs. Using these human lung organoids, we identified the viral cell tropism, investigated early cell response to viral infection, and demonstrated human lung organoids as a platform for anti-viral therapeutic drugs discovery.

As to viral cell tropism, club cells are identified as a new type of SARS-CoV-2 target cells. Ciliated and AT2 cells are previously reported target cells and are also confirmed in this study. As we know, the lower respiratory tract starts with trachea, extends to bronchi and bronchioles, and terminates in alveoli. From the proximal to distal airway, the number of ciliated cells decreases and the number of club cells increases (Rock et al., 2010; Bustamante-Marin and Ostrowski, 2017). And AT2 cells reside in alveoli. Therefore, our finding strongly suggests a potential model for virus transmission that SARS-CoV-2 sequentially infects ciliated, club and AT2 cells along the upper airway down to alveoli.

More importantly, we discovered that the metabolic processes were significantly downregulated upon SARS-CoV-2 infection. Generally, most viruses examined to date promote aerobic glycolysis, nucleotide and lipid synthesis as well as glutaminolysis, which is believed to provide specific substrates and energy for virus replication and virus particles assembly (Sanchez and Lagunoff, 2015; Thaker et al., 2019). More and more evidence also indicate that each virus species can reprogram unique metabolism pathways. For example, lipid synthesis is generally increased in most viruses infected cells, but several phospholipid species and lipid classes (e.g., sphingomyelin, TAG) are decreased in hepatitis C virus (HCV) infected cells (Diamond et al., 2010; Heaton and Randall, 2011; Ketter and Randall, 2019). It is worth noting that a single virus can induce different metabolic changes in different host cell types. For example, glucose uptake is induced in HIV-1 infected CD4^+^ T cells, whereas it is substantially reduced in infected macrophages (Hollenbaugh et al., 2011). This observation strongly demonstrates that the reprogramming of specific metabolic pathways is cell type dependent and further highlights the importance of the infected cell type tested for metabolic investigation. This phenomenon also reminds us that most virus-associated metabolomics studies are done in immortalized tumor cells and by chance the significantly altered metabolic pathways upon virus infection, including glycolysis, fatty acid synthesis and glutaminolysis, are often similarly activated in many cancer cells (Kroemer and Pouyssegur, 2008). Therefore, it is pivotal to use normal or primary cells to explore virus-induced metabolism reprogramming in order to reach more physiopathological relevant conclusions. It is known that metabolism *in vivo* is quite different from that found *in vitro* in cell cultures. A current report shows that diacylglycerols (DAG) is reduced in COVID-19 patients' plasma (Song et al., 2020), which is consistent with our results that the key enzymes MOGAT1/2/3 for DAG synthesis are downregulated (Fig. S4B). In this scenario, our human lung organoids can serve as a better platform than currently used tumor cells to investigate cellular metabolism. In this study, we discover a downregulation of cellular metabolism upon SARS-CoV-2 infection, which is a unique feature compared to most tested viruses. However, more questions remain open, such as how SARS-CoV-2 regulate cellular metabolism and how the decreased metabolism regulates SARS-CoV-2 replication, assembly, egress and even pathogenesis.

It has been reported that Spike (S) proteins of SARS-CoV and SARS-CoV-2 induce shedding of ACE2 via TMPRSS2 or ADAM17, which is a crucial mechanism for virus-induced pathogenesis (Kuba et al., 2005; Glowacka et al., 2010; Heurich et al., 2014; Banu et al., 2020; Vaduganathan et al., 2020; Verdecchia et al., 2020; Xiao et al., 2020). In the current study, the mRNA expression level of ACE2 was also found significantly decreased after SARS-CoV-2 infection. Thus, SARS-CoV-2 is able to downregulate its receptor ACE2 via multiple mechanisms. Full dissection of these mechanisms might reveal more information about viral pathogenesis as well as identify potential therapeutic targets to treat COVID-19.

We noted that remdesivir reduced viral load to about 1/10 but reduced infectious virus titer to less than 1/1000. Similar phenomena, with potent inhibitory effect on virus titer and much less effect on viral load, have been reported in remdesivir-treated rhesus macaques with SARS-CoV-2 infection (Williamson et al., 2020). An explanation for the phenomena might be that virus particles with RNA containing the remdesivir-metabolized adenine analogue are defective for infection, in addition to the known mechanism that remdesivir induces delayed chain termination (Eastman et al., 2020).

In summary, we demonstrated that hESCs-derived lung organoids could serve as a pathophysiological model to investigate the underlying mechanism of SARS-CoV-2 infection and to discover and test therapeutic drugs for COVID-19.

## MATERIALS AND METHODS

### Maintenance of human ESCs

All experiments in the present study were performed on H9 human embryonic stem cells (hESCs). hESCs were maintained in feeder-free culture conditions in 6-well tissue culture dishes on Matrigel (BD Biosciences, 354277) in mTeSR1 medium (Stem Cell Technologies, 05850) at 37 °C with 5% CO_2_. Cells were passaged with TrypLE (Gibco) at 1:6 to 1:8 split ratios every 4 days.

### Generation of hESCs derived hAWO and hALO

hESCs derived hAWOs and hALOs were generated as previously described with modifications (McCauley et al., 2017; Yamamoto et al., 2017; Chen et al., 2018). H9 cells (~90% confluence) were cultured in 24-well tissue dishes for 3 days in RPMI1640 medium supplemented with 100 ng/mL Activin A (R&D Systems, 338-AC-050) and 2 μmol/L CHIR99021 (Tocris, 4423-10MG), followed by 4 days with 200 ng/mL Noggin (R&D Systems, 6057-NG-100), 500 ng/mL FGF4 (Peprotech, 100-31-1MG), 2 μmol/L CHIR99021 and 10 μmol/L SB431542 (Tocris, 1614-10MG) in Advanced DMEM/F12 (Life Technologies, 12634010). After 7 days' treatment with above-mentioned factors, anterior foregut endodermal cells were embedded in a droplet of Matrigel (BD Biosciences, 356237) and incubated at 37 °C with 5% CO_2_ for 20–25 min. After matrigel solidification, cells were then fed with 20 ng/mL human BMP4 (R&D Systems, PRD314-10), 0.5 μmol/L all-trans retinoic acid (ATRA, Sigma-Aldrich, R2625), 3.5 μmol/L CHIR in DMEM/F12 (Life Technologies, 11320033) with 1% Glutamax (Gibco, 35050061), 2% B27 supplement (Life Technologies, 17504044) basal medium from day 8 to day 14. For preconditioning toward lung progenitor stem cell differentiation, NKX2-1^+^ VAFE-enriched cells were cultured in the same basal medium supplemented with 3 μmol/L CHIR99021, 10 ng/mL human FGF10 (R&D Systems, 345-FG-025), 10 ng/mL human KGF (novoprotein, CM88) and 20 μmol/L DAPT (Sigma, D5942) from day 14 to day 21. From day 21, human airway organoids (hAWOs) medium was prepared from Ham's F12 (Gibco, 21127022) by supplementation with 50 nmol/L dexamethasone (Sigma-Aldrich, D4902), 100 nmol/L 8-Br-cAMP (Biolog Life Science Institute, B007-500), 100 nmol/L 3-isobutyl-1-methylxanthine (Wako, 095-03413), 10 ng/mL KGF, 1% B-27 supplement, 0.25% BSA (Sigma, A1470) and 0.1% ITS premix (Corning, 354351). And human alveolar organoids (hALOs) medium was prepared by supplementing 3 μmol/L CHIR99021 and 10 μmol/L SB431542 to the human airway organoids medium. Organoids were transferred into new Matrigel droplets every 4–7 days using mechanical digestion.

### Quantitative RT-PCR

Total RNA was extracted using the Trizol reagent (MRC, TR1187) and cDNA was converted from 1μg total RNA using the ReverTraAce Kit (TOYOBO, 34520B1). The qPCR reactions were done on Roche LightCycler® 96 PCR system with the SYBR Premix Ex Taq™ Kit (TAKARA, RR420A). Gene expression levels were normalized to *GAPDH* and compared to gene expression levels in hESCs. Three or more biological replicates were performed for each assay and data bars represent mean ± SD. Primers used in this study are listed in Table S1.

### SARS-CoV-2 infection, drug test, and virus titers determination

SARS-CoV-2 (WIV04) (Zhou et al., 2020b) was propagated 7 times on Vero E6 cells in DMEM (Gibco, C12430500BT) with 2% FBS (Gibco, 10099-141) at 37 °C with 5% CO_2_. The SARS-CoV-2 isolate was obtained and titrated by plaque assay on Vero E6 cells. Human airway and alveolar organoids were harvested, sheared and resuspended in Ham's F12 medium (Gibco, 21127022) and infected with virus at multiplicity of infection (MOI) of 1. After 2 h of SARS-CoV-2 virus adsorption at 37 °C in the incubator, cultures were washed twice with Ham's F12 medium to remove unbound viruses. hAWOs and hALOs were re-embedded into Matrigel (BD Biosciences, 356237) in 24-well tissue plates, and cultured in 500 μL corresponding organoid media, respectively. In drug testing experiments, different drugs at concentration of 10 μmol/L were added to the culture 2 h after virus infection. As for effective drug-remdesivir, hAWOs and hALOs were treated with different doses, which were mentioned as above. Samples were harvested at indicated time points by collecting the supernatant in the wells and the cells via resuspending the matrigel droplet containing organoids into 500 μL Ham's F12 medium. The viral RNA in the supernatants was extracted by Magnetic Beads Virus RNA Extraction Kit (Shanghai Finegene Biotech, FG438). The intracellular RNA was extracted with Trizol reagent (Invitrogen, 15596026). The viral RNA was quantified by real-time qPCR with Taqman probe targeting the RBD region of S gene. Viral titers (TCID50 equivalents per mL) were determined by plaque assay on Vero E6 cells.

### Evaluation of antiviral activities of the remdesivir and neutralizing antibodies

To evaluate the antiviral efficacy of remdesivir and neutralizing antibody CB6, hAWOs and hALOs were pre-infected with SARS-CoV-2 (MOI of 1) for 2 h, and the virus-drug or virus-neutralizing antibody mixtures were subsequently added to the culture for 48 h. The cell supernatants were collected and viral titers (TCID50 equivalents per mL) were determined by plaque assay on Vero E6 cells. The cytotoxicity of remdesivir to organoids was determined by viable cell counting. Organoids were harvested by incubation with 0.25% Trypsin-EDTA (Gibco, 25200072) for 30 min at 37 °C until single cell suspension were achieved. 20 μL samples were mixed with 20 μL 0.4% trypan blue (Sigma, T8154) by gently pipetting, and then 20 μL of the mixtures were loaded into chamber of the hemocytometers. Counts were performed by triplicate according to the standard methodology.

### RNA-seq sequencing and data analysis

Total RNA in the cells was extracted using Trizol (Invitrogen, 15596026) according to the manufacturer's protocol, and 1 μg RNA was used to reverse transcribed into cDNA using Oligo (dT). Fragmented RNA (average length approximately 200 bp) was subjected to first strand and second strand cDNA synthesis followed by adaptor ligation and enrichment with a low-cycle according to the instructions of NEBNext” UltraTM RNA Library Prep Kit for Illumina (NEB, USA). The purified library products were evaluated using the Agilent 2200 TapeStation and Qubit”2.0 (Life Technologies, USA).

Reads were aligned to the human reference genome hg38 with bowtie2 (Langmead and Salzberg, 2012), and RSEM (Li and Dewey, 2011) was used to quantify the reads mapped to each gene. Gene expression was normalized by EDASEQ (Risso et al., 2011). Differentially expressed genes were obtained using DESeq2 (version 1.10.1) (Love et al., 2014), a cutoff of Q-value < 0.05 and log2 (fold-change) > 1 was used for identify differentially expressed genes. All differentially expressed mRNAs were selected for GO analyses cluster Profiler (Yu et al., 2012). Other analysis was performed using glbase (Hutchins et al., 2014). The RNA-seq supporting this study is available at GEO under GSE155717.

### Immunofluorescence staining

For immunofluorescence staining, samples were transferred into 1.5 mL tubes and fixed with 4% paraformaldehyde overnight at 4 °C or 2 h. Following fixation, paraformaldehyde was removed the organoids were rinsed three times with PBS, then the samples were overlaid with O.C.T compound and frozen in liquid nitrogen. The frozen samples were cryosectioned into 6 μm sections, washed with PBS three times and permeabilized with 0.2% Triton X-100 (Sigma, T9284)/PBS for 20 min at RT, rinsed again with PBS and then blocked with 5% BSA at RT for 1 h. The samples were incubated with primary antibodies overnight at 4 °C, and then stained with secondary antibodies at RT for 40 min. Nuclear counterstained with DAPI (Sigma, D9542) for 3 min, then covered with glass microscope slides and imaged with the Nikon A1 confocal microscope. NIS-Elements software was used to render Z-stack three-dimensional images. The primary and secondary antibodies used in this study are listed in Table S2.

### Whole-mount immunofluorescence

For whole-mount immunofluorescence staining, Cell Recovery Solution (Corning, 354253) was used to isolate organoids from matrigel. Samples were transferred into the glass bottom microwell dishes (Corning, P35G-0-20-C), fixed with 4% paraformaldehyde overnight at 4 °C or 2 h at RT, washed with PBS three times, permeabilized and blocked with 0.2% Triton X-100 and 5% BSA in PBS at RT for 1 h. Primary antibodies were incubated overnight at 4 °C, and then stained with secondary antibodies at RT for 45 min. Nuclear counterstained with DAPI for 5 min. Organoids were mounted and imaged using the Nikon A1 confocal microscope. The images were processed using NIS-Elements software for the 3D reconstruction. The primary and secondary antibodies used are listed in Table S2.

### Transmission electron microscopy

Organoids were collected and fixed in 2.5% glutaraldehyde for 24 h, washed with 0.1 mol/L Phosphate buffer (19 mL 0.2 mol/L NaH_2_PO_4_, 81 mL 0.2 mol/L Na_2_HPO_4_) for 3 times, and further fixed with 1% osmium tetroxide for 2 h at room temperature. The fixed organoids were then washed with phosphate buffer and dehydrated with 30%, 50%, 70%, 80%, 85%, 90%, 95%, and 100% alcohol sequentially. After a step of infiltration with different mixtures of acetone-epon (2:1, 1:1, *v*/*v*), the samples were embedded in pure Epon. Polymerization was performed by incubation at 60 °C for 48 h. Ultra-thin sections (80–100 nm) were cut on Ultramicrotome (Leica EM UC7), put on grids and stained with uranyl acetate and lead citrate. After wash and drying, images were acquired by the digital camera on TEM (FEI, Tecnai G2 20 TWIN, 200 kv), with identical magnificence.

### Experimental replicates and statistical analysis

Error bars in these figures indicate S.D. (for qRT-PCR) and S.E.M (for other assays). Unpaired, two-tailed Student's *t* tests were used for comparisons between two groups of *n* = 3 or more samples. *P* < 0.05 was defined as statistical significance. Immunofluorescence (IF) imaging were done on Z-stacks acquired with confocal microscope at least three (*n* = 3) independent biological samples or more. The co-localization of quantitative analysis of specific immunofluorescence marker was shown in figure legends. All of the statistical analyses in this study were done with GraphPad Prism 8 software.

## Electronic supplementary material

The online version of this article (https://doi.org/10.1007/s13238-020-00811-w) contains supplementary material, which is available to authorized users.

## Supplementary Material

13238_2020_811_MOESM1_ESM **Supplementary Figure S1. SARS-CoV-2 dose not infect basal cells, goblet cells or alveolar type I cells**. (**A,B**) Representative immunofluorescence images of nucleoprotein, ACE2 and indicated cell linage marker expression with DNA stain (DAPI). Basal cells (P63^+^) and goblet cells (MUC5AC^+^) were stained in human airway organoids at indicated time points (**A**). Alveolar type I cells (PDPN^+^) were stained in human alveolar organoids (**B**). Scale bar, 100µm; bottom left corner, 20µm. Boxes represent zoom views.

## ABBREVIATIONS

AFE, anterior foregut endoderm; Apo, apolipoproteins; AT2, alveolar type 2; CC_50_, halfcytotoxic concentration; CMs, convoluted membranes; COVID-19, coronavirus disease 2019; DE, definitive endoderm; DGATs, diacylglycerol acyltransferases; DMVs, double membrane vesicles; EC_50_, half-maximal effective concentration; ERGIC, endoplasmic reticulum-Golgi intermediate compartment; ESCs, embryonic stem cells; FABPs, fatty acid-binding proteins; FXR, farnesoid X receptor; hAWOs, human airway organoids; hALOs, human alveolar organoids; hESCs, human embryonic stem cells; hLOs, human lung organoids; LIPINs, lipin phosphatidic acid phosphatases; LPs, lung progenitors; LXRα, liver X receptor α; MaREA, metabolic reaction enrichment analysis; MOGATs, Monoacylglycerol acyltransferases; MOI, multiplicity of infection; NP, viral nucleocapsid protein; RXR, retinoid X receptor; SARS-CoV-2, severe acute respiratory syndrome coronavirus 2; SLC, the solute carrier; TAG, triacylglycerols; TEM, transmission electron microscopy; VAFE, ventralized anterior foregut endoderm
