# Supplementary material for: Host metabolism dysregulation and cell tropism identification in human airway and alveolar organoids upon SARS-CoV-2 infection
Source: Protein Cell. 2020 Dec 12;12(9):717–33. doi: 10.1007/s13238-020-00811-w (PMC7732737; doi:10.1007/s13238-020-00811-w)
Supplement: 13238_2020_811_MOESM1_ESM — Supplementary Figure S1. SARS-CoV-2 dose not infect basal cells, goblet cells or alveolar type I cells. (A,B) Representative immunofluorescence images of nucleoprotein, ACE2 and indicated cell linage marker expression with DNA stain (DAPI). Basal cells (P63+) and goblet cells (MUC5AC+) were stained in human airway organoids at indicated time points (A). Alveolar type I cells (PDPN+) were stained in human alveolar organoids (B). Scale bar, 100µm; bottom left corner, 20µm. Boxes represent zoom views. [file 13238_2020_811_moesm1_esm.pdf]

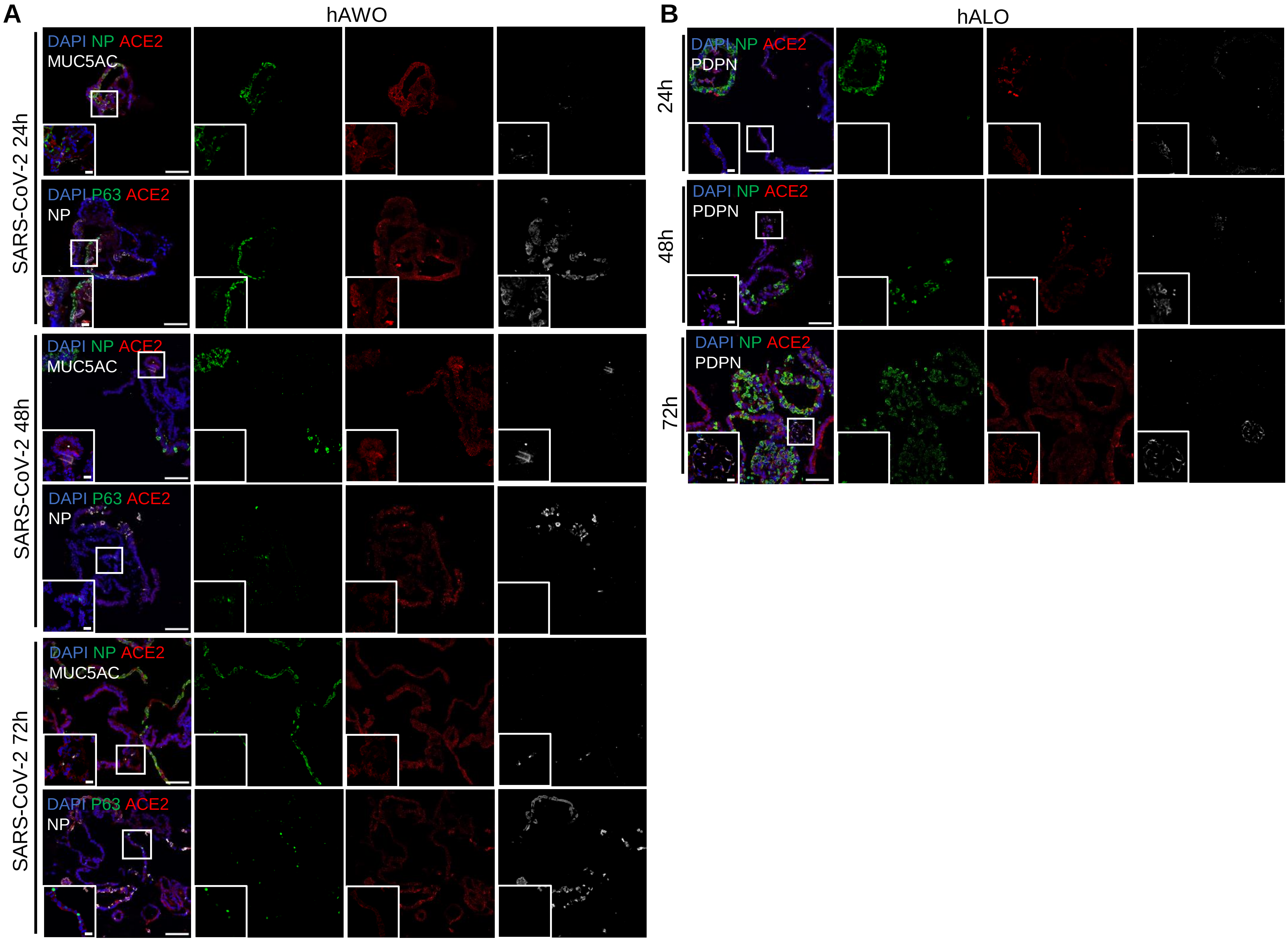

Supplementary Figure S1. SARS-CoV-2 dose not infect basal cells, goblet cells or alveolar type 1 cells

Supplementary Figure S1. SARS-CoV-2 dose not infect basal cells, goblet cells or alveolar type I cells. (A,B) Representative immunofluorescence images of nucleoprotein, ACE2 and indicated cell linage marker expression with DNA stain (DAPI). Basal cells ($P63^{+}$) and goblet cells ($MUC5AC^{+}$) were stained in human airway organoids at indicated time points **(A)**. Alveolar type I cells ($PDPN^{+}$) were stained in human alveolar organoids **(B)**. Scale bar, 100µm; bottom left corner, 20µm. Boxes represent zoom views.

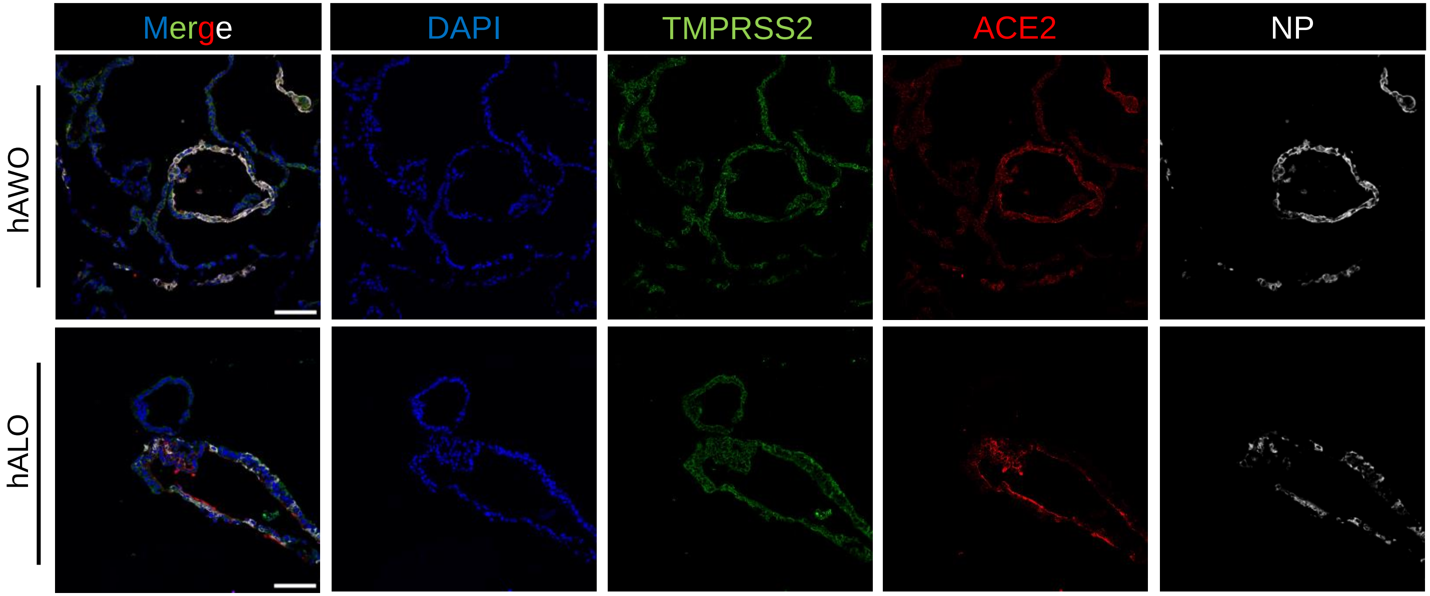

Supplementary Figure S2. TMPRSS2 is ubiquitously expressed in human airway and alveolar organoid cells. Immunofluorescence images of SARS-CoV-2 infected human airway and alveolar organoids. TMPRSS2 (green) is broadly expressed in almost all human lung epithelial cells. Virus infected cells (nucleoprotein positively) highly express ACE2 (red). Scale bar, 100μm.

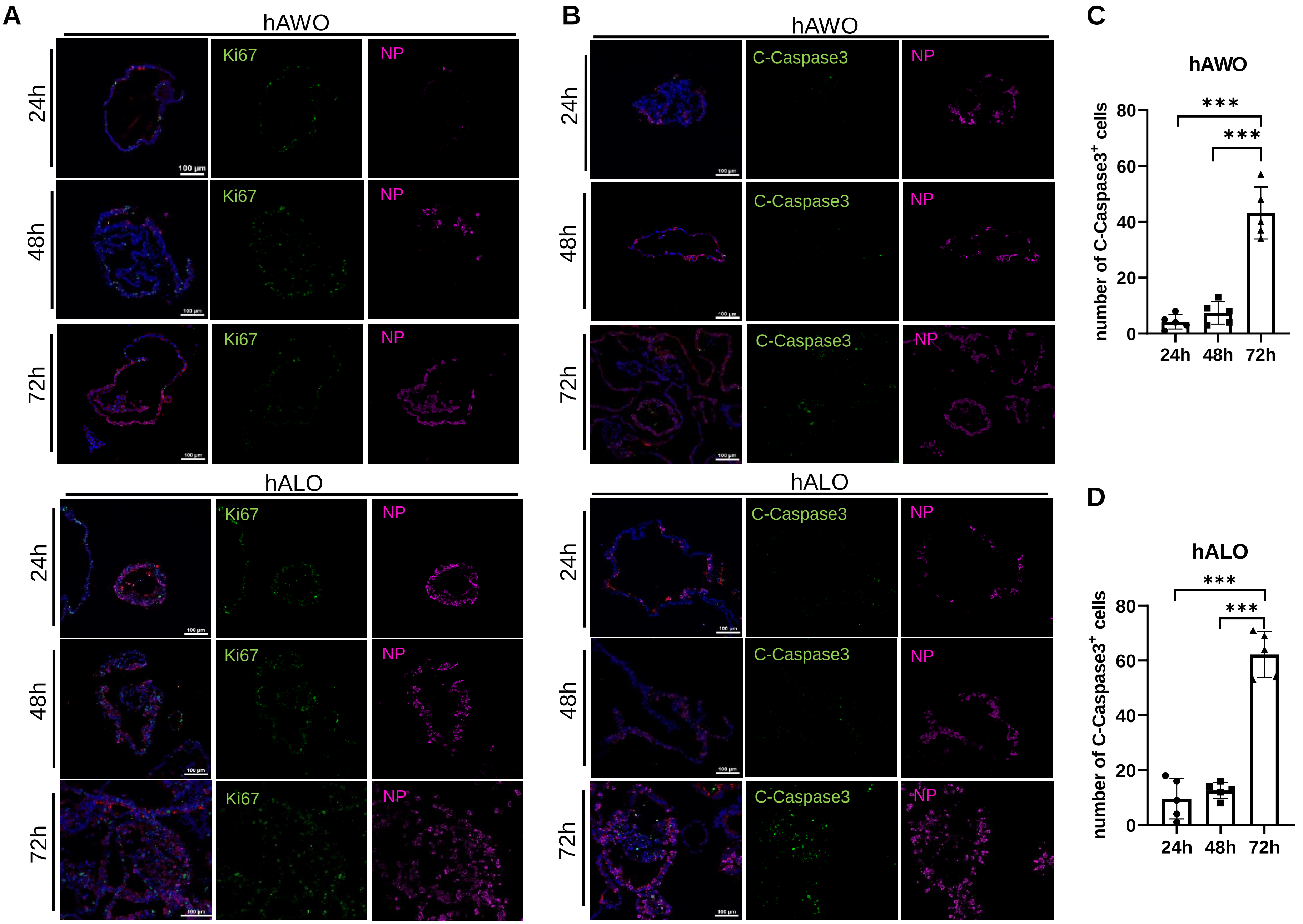

Supplementary Figure S3. SRAS-CoV-2 infection induces apoptosis in human airway and alveolar organoids

Supplementary Figure S3. SRAS-CoV-2 infection induces apoptosis in human airway and alveolar organoids. (A) SARS-CoV-2 infected human airway and alveolar organoids are stained by cell proliferation marker, Ki67 (green) at 24, 48 and 72 hpi. Scale bar, 100µm. **(B)** Long term infection of SARS-CoV-2 induces apoptosis. Cleaved caspase-3 (green) were observed within virus infected organoids at 72 hpi. Scale bar, 100µm. **(C,D)** Number of cleaved caspase-3 positive cells in SARS-CoV-2 infected human airway organoids **(C)** and human alveolar organoids **(D)**. n=5 organoids per condition. *** $p < 0.001$ by unpaired, two-tailed Student's t test.

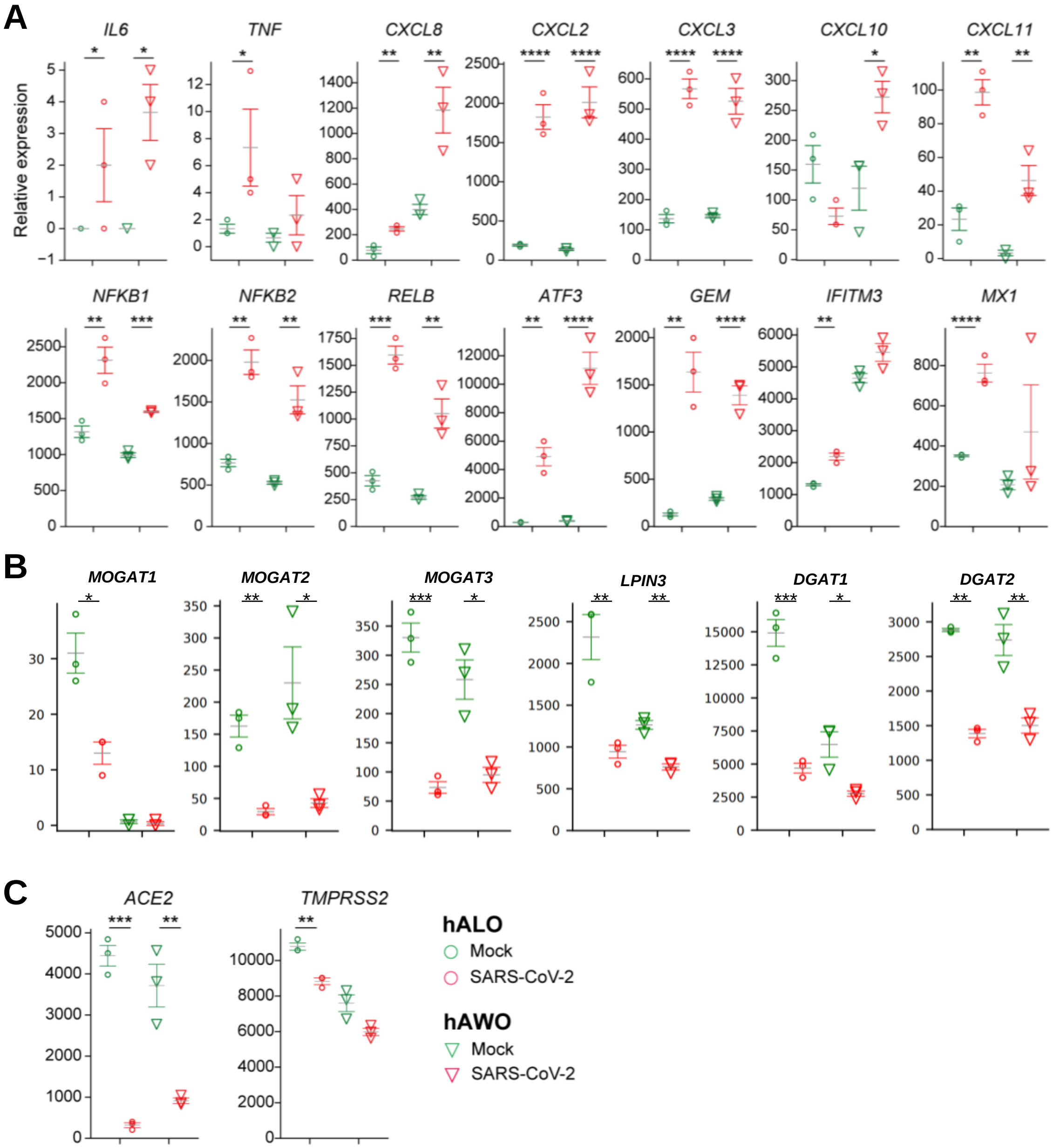

Supplementary Figure S4. Differentially expressed genes in the SARS-CoV-2-infected human lung organoids

D

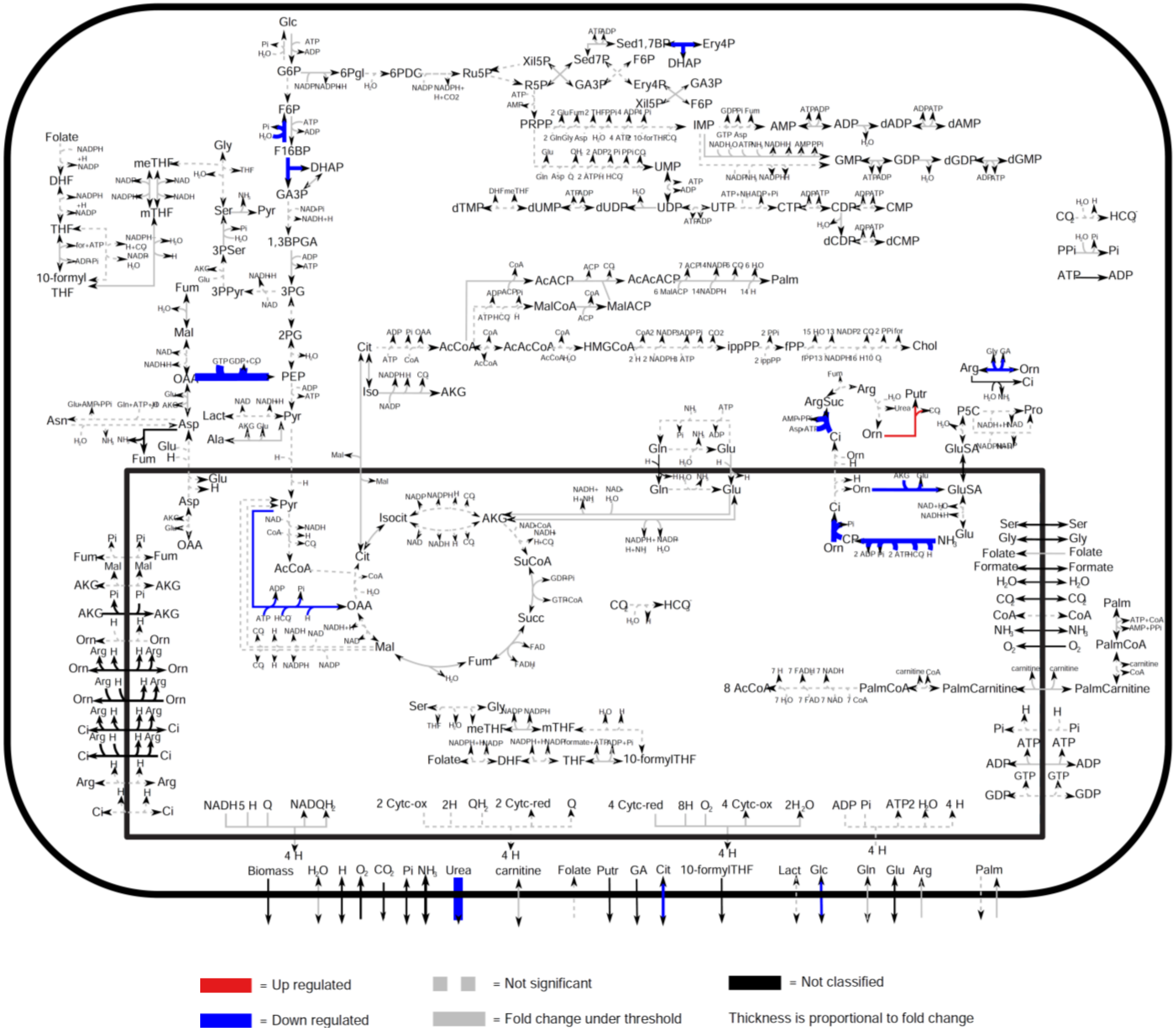

Supplementary Figure S4. Differentially expressed genes in the SARS-CoV-2-infected human lung organoids

E

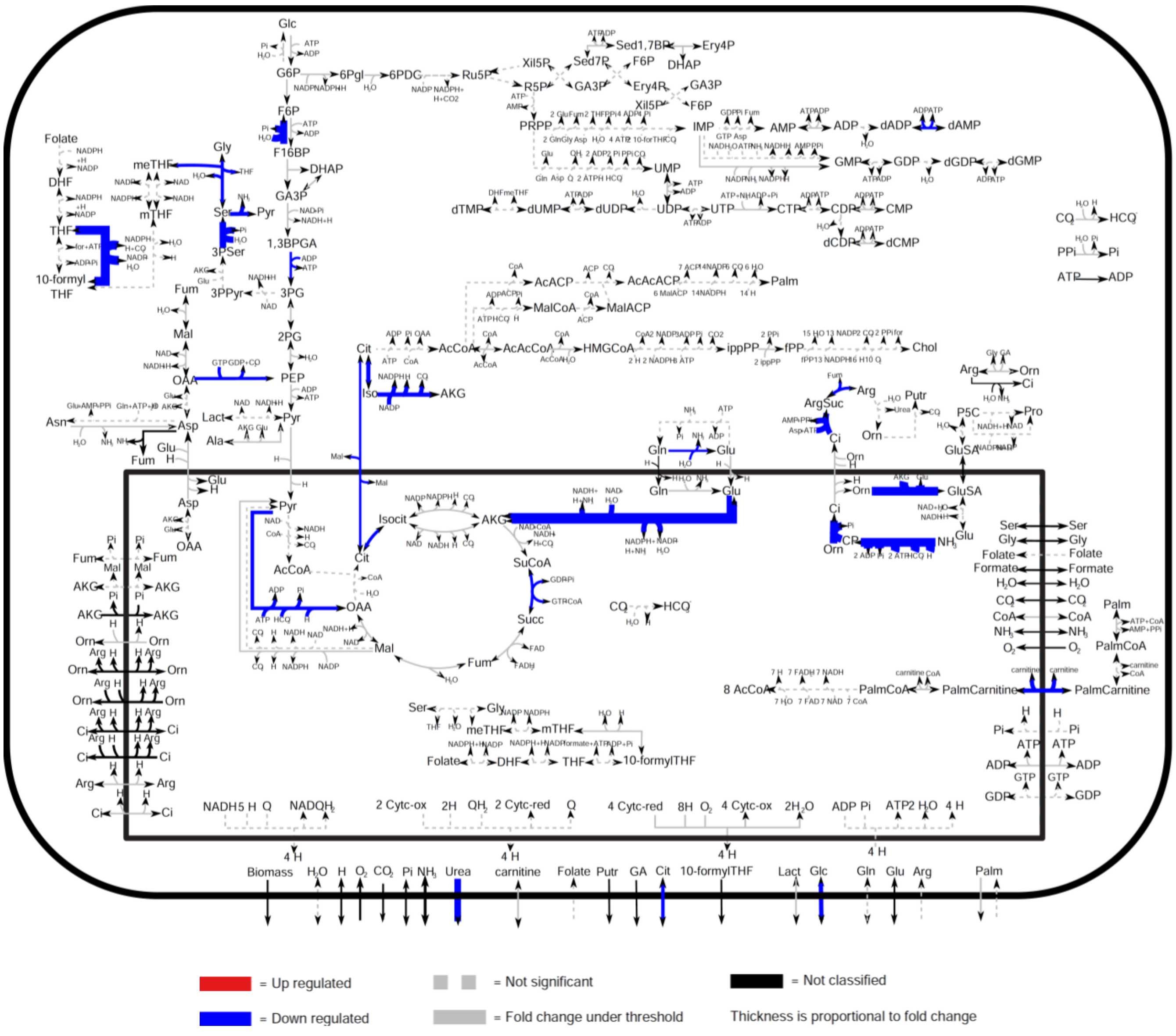

Supplementary Figure S4. Differentially expressed genes in the SARS-CoV-2-infected human lung organoids

Supplementary Figure S4. Differentially expressed genes in the SARS-CoV-2-infected human lung organoids. (A) Expression level of immune response related genes at 48 hpi. **(B)** Expression level of critical enzymes catalyzing triacylglycerol synthesis. **(C)** Expression level of ACE2 and TMPRSS2. **(D)** Metabolism change analysis revealed downregulated urea cycle in virus infected hAWOs by the MaRE4Galaxy tool. **(E)** Metabolism change analysis revealed downregulated folate metabolism, glutamine metabolism and urea cycle in virus infected hALOs by the MaRE4Galaxy tool. For **A-C**, The grey lines are the means of the three biological replicates, and the error bars are the standard error of the mean. Dare expressed as normalized read counts. P-values are from a one-tailed Student's t test. * $p<0.05$, ** $p<0.01$, *** $p<0.001$, **** $p<0.0001$.

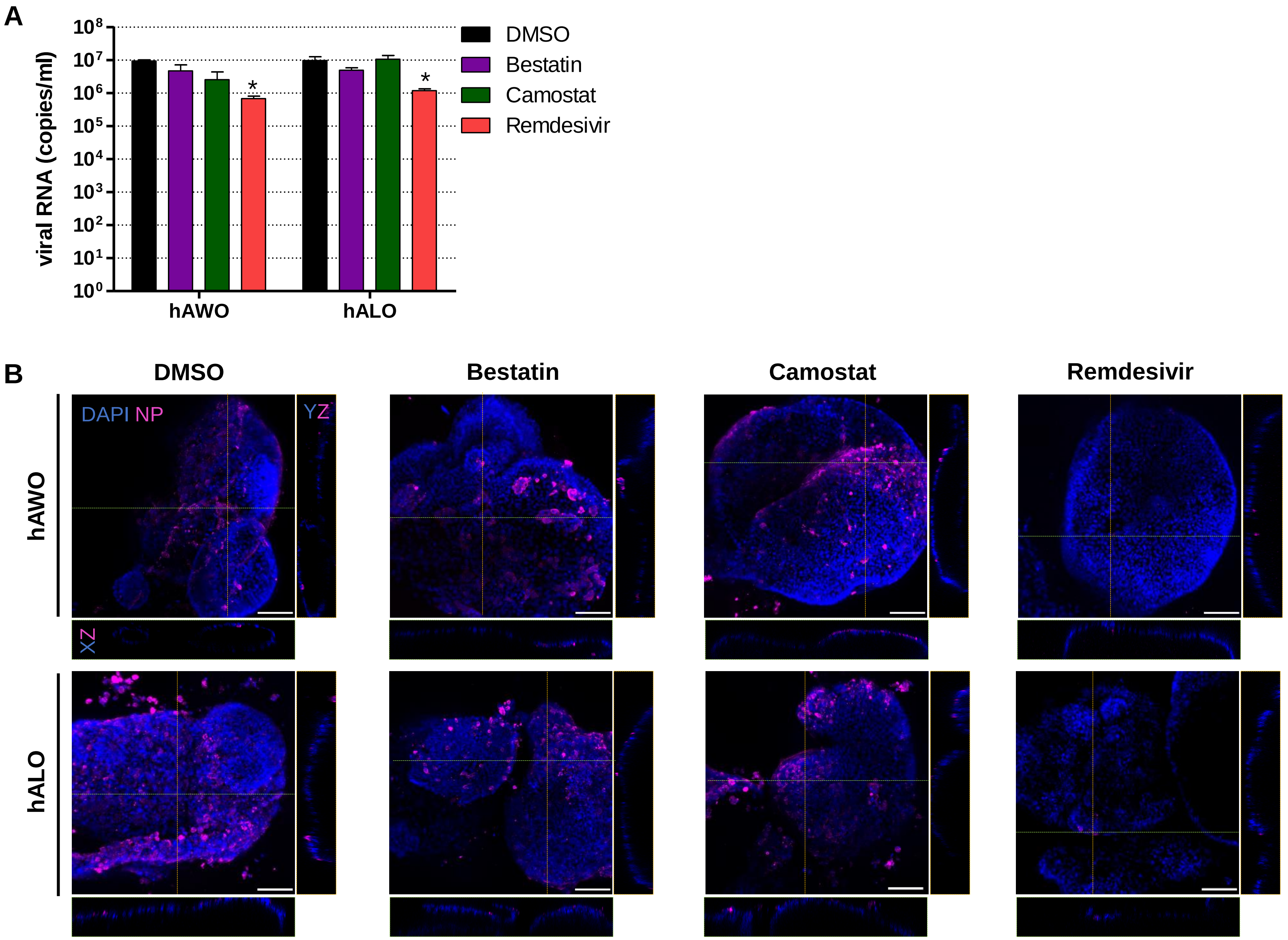

Supplementary Figure S5. Remdesivir inhibits SARS-CoV-2 replication in human lung organoids.

Supplementary Figure S5. Remdesivir inhibits SARS-CoV-2 replication in human lung organoids. (A) hAWOs and hALOs were infected with SARS-CoV-2, the indicated compounds were added into the culture media at 2hpi. The viral RNA in the culture supernatant was determined by qRT-PCR after 48h. **(B)** Whole-mount staining of human airway and alveolar organoids, which were harvested 48h after drug treatment. Scale bars: 100 µm.

Table S1. Primers for qRT-PCR.

Gene	Forward primer (5’ to 3’)	Reserve primer (5’ to 3’)
GAPDH	ACAACTTTGGTATCGTGGAAGG	GCCATCACGCCACAGTTTC
POU5F1	GGGAGATTGATAACTGGTGTGTT	GTGTATATCCCAGGGTGATCCTC
FOXA2	GGAGCAGCTACTATGCAGAGC	CGTGTTCATGCCGTTCATCC
SOX2	TACAGCATGTCCTACTCGCAG	GAGGAAGAGGTAACCACAGGG
SOX9	AGCGAACGCACATCAAGAC	CTGTAGGCGATCTGTTGGGG
SOX17	GTGGACCGCACGGAATTTG	GGAGATTCACACCGGAGTCA
NKX2.1	CTCATGTTCATGCCGCTC	GACACCATGAGGAACAGCG
P63	CCACCTGGACGTATTCCACTG	TCGAATCAAATGACTAGGAGGGG
MUC5AC	ACCAATGCTCTGTATCCTTCCC	GTTTGGGTGGAGTAAGCCACA
SFTPC	AGCAAAGAGGTCCTGATGGA	CGATAAGAAGGCGTTTCAGG
SCGB1A1	TTCAGCGTGTCATCGAAACCC	ACAGTGAGCTTTGGGCTATTTTT
ACE2	CAAGAGCAAACGGTTGAACAC	CCAGAGCCTCTCATTGTAGTCT
TMPRSS2	GCAGTGGTTTCTTTACGCTGT	CCGCAAATGCCGTCCAATG
Viral RNA PCR primer	CAATGGTTTAACAGGCACAGG	CTCAAGTGTCTGTGGATCACG
Viral RNA PCR probe	ACAGCATCAGTAGTGTCAGCAATGTCTC	

Table S2. Antibody list

Primary Antibodies	Dilution rate	Manufacturer	Cat. No.
NKX2.1	1:250	Abcam	ab76013
SOX2	1:1000	Abcam	AB97959
SOX9	1:40	R&D systems	AF3075
P63	1:200	Abcam	ab124762
MUC5AC	1:150	Thermo Fisher Scientific	MA5-12178
CC10	1:300	Abcam	Ab40873
SFTPC	1:300	SEVEN HILLS	WRAB-76694
AQP5	1:150	Abcam	ab92320
PDPN	1:200	Abcam	ab10288
acetylated Tubulin	1:1000	Sigma	T7451
Pro-SPC	1:200	EMD-Millipore	#AB3786
E-CAD	1:100	R&D systems	AF748
Ki67	1:250	Abcam	Ab1667
Cleaved Caspase-3	1:400	Cell Signaling Technology	#9661
Human ACE-2	1:100	R&D systems	AF933
TMPRSS2	1:150	Abcam	Ab109131
SARS-CoV-2 Nucleocapsid	1:200	Sino biological	40143-MM08
SARS-CoV-2 Nucleocapsid	1:5000	Kindly provide by Prof. Zheng-Li Shi	Reference(Zhou et al., 2020)
Dnokey anti-goat (RRX)	1:500	Jackson ImmunoResearch	705-295-147

Donkey anti-rabbit (Alexa488)	1:500	Thermo Fisher Scientific	A-21206
Donkey anti-mouse (Alexa647)	1:300	Thermo Fisher Scientific	A-31571

Zhou, P., Yang, X.L., Wang, X.G., Hu, B., Zhang, L., Zhang, W., Si, H.R., Zhu, Y., Li, B., Huang, C.L., *et al.* (2020). A pneumonia outbreak associated with a new coronavirus of probable bat origin. Nature 579, 270-273.
